# *Wnt9a* deficiency discloses a repressive role of Tcf7l2 on endocrine differentiation in the embryonic pancreas

**DOI:** 10.1038/srep19223

**Published:** 2016-01-14

**Authors:** G. Pujadas, S. Cervantes, A. Tutusaus, M. Ejarque, L. Sanchez, A. García, Y. Esteban, L. Fargas, B. Alsina, C. Hartmann, R. Gomis, R. Gasa

**Affiliations:** 1 Diabetes and Obesity Research Laboratory, Institut d’Investigations Biomèdiques August Pi i Sunyer, Barcelona, Spain; 2 Centro de Investigación Biomédica en Red de Diabetes y Enfermedades Metabólicas Asociadas, Barcelona, Spain; 3 University of Barcelona, Barcelona, Spain; 4 Departament de Ciències Experimentals i de la Salut, Facultat de la Salut i de la Vida, Universitat Pompeu Fabra, Barcelona, Spain; 5 Dept. of Bone and Skeletal Research, Institute of Experimental Musculoskeletal Medicine (IEMM), University Hospital Muenster, Muenster, Germany

## Abstract

Transcriptional and signaling networks establish complex cross-regulatory interactions that drive cellular differentiation during development. Using microarrays we identified the gene encoding the ligand Wnt9a as a candidate target of Neurogenin3, a basic helix-loop-helix transcription factor that functions as a master regulator of pancreatic endocrine differentiation. Here we show that Wnt9a is expressed in the embryonic pancreas and that its deficiency enhances activation of the endocrine transcriptional program and increases the number of endocrine cells at birth. We identify the gene encoding the endocrine transcription factor *Nkx2-2* as one of the most upregulated genes in *Wnt9a*-ablated pancreases and associate its activation to reduced expression of the Wnt effector Tcf7l2. Accordingly, *in vitro* studies confirm that Tcf7l2 represses activation of *Nkx2-2* by Neurogenin3 and inhibits *Nkx2-2* expression in differentiated β-cells. Further, we report that Tcf7l2 protein levels decline upon initiation of endocrine differentiation *in vivo*, disclosing the downregulation of this factor in the developing endocrine compartment. These findings highlight the notion that modulation of signalling cues by lineage-promoting factors is pivotal for controlling differentiation programs.

In the developing pancreas, multipotent pancreatic progenitors give rise to endocrine (α, β, δ, PP and ε) and exocrine (acinar and ductal) cell lineages through the sequential and coordinated activation-inhibition of a number of transcription factors. The transcription factor Neurogenin3 (Neurog3) functions as a master regulator of endocrine cell development. In the mouse, the peak of *Neurog3* expression occurs between embryonic days (E)13.5 and 16.5, a stage known as the secondary transition when major pancreatic differentiation waves take place. Expression of Neurog3 instructs bipotent duct/endocrine progenitors located in the trunk domain of the pancreatic epithelium to adopt an endocrine cell fate. Neurog3 functions as a potent transcriptional activator that initiates the endocrine transcriptional program by inducing the expression of several transcription factors, including *Pax4, Nkx2-2, NeuroD1* or *Insm1*, which will carry on the differentiation process (for extensive reviews see^1^,^2^).

The canonical Wnt pathway is transduced through stabilized (dephosphorylated) β-catenin protein that translocates to the nucleus and cooperates with lymphoid-enhancer-binding factor T-cell factor (LEF/TCF) DNA-binding proteins to activate Wnt-dependent genes. There is evidence to suggest that canonical Wnt participates in pancreas organogenesis, including the dynamic regulation of canonical Wnt ligands and receptors within the developing pancreas^3^,^4^, the presence of dephosphorylated β-catenin in the pancreatic epithelium from E11.5 to E15.5^4^,^5^,^6^ and the identification of cells positive for Wnt reporter gene expression in embryonic pancreases of mouse reporter lines^7^,^8^. At early stages, activation of Wnt has been shown to prevent pancreas development, possibly through inhibition of organ specification^3^,^9^. Later, Wnt/β-catenin is needed for the proliferation of pancreatic progenitors and proper organ growth. Thus, ablation of β-catenin^5^,^6^ or the ligand Wnt7b^10^ and transgenic expression of Wnt signalling inhibitors^4^ result in severe organ hypoplasia whilst, conversely, β-catenin stabilization leads to increased organ size^9^. More disputed is the role of Wnt signals in the differentiation of the pancreatic lineages. While some studies ascribe to this pathway an exclusive role in exocrine development^5^,^6^,^9^, others indicate additional roles in the endocrine compartment^4^,^7^. In support of the participation of Wnt signals in endocrine development, expression profile data from isolated Neurog3+ cells and gain-of-function models identified the Wnt pathway, amongst other intercellular signalling pathways, as a potential target of Neurog3^11^,^12^. To date, only non-canonical planar cell polarity/Wnt signalling has been connected to endocrine differentiation downstream of Neurog3^11^.

Using gene expression profiling in a Neurog3-dependent endocrine differentiation cell model^13^, we identified the gene encoding the Wnt ligand Wnt9a, formerly Wnt14, as a potential Neurog3 target. Wnt9a signalling has been shown to participate in joint formation where it influences cell fate assignment by suppressing chondrocyte differentiation from bi-potential chondro-synovio progenitors^14^,^15^,^16^,^17^. In the zebrafish, the Wnt9a homolog was identified in the central nervous system, pro-nephric ducts and sensory organs and connected to palate development^18^,^19^. In the chicken, Wnt9a has been implicated in hepatic epithelium morphogenesis and in the development of the retina^20^. However, there are no prior reports on Wnt9a in the pancreas. Herein, we present *in vitro* and *in vivo* evidence of the participation of Wnt9a-dependent signalling in islet cell development. We show that *Wnt9a* loss increases the formation of pancreatic endocrine cells and present the Wnt effector Tcf7l2 as a likely mediator of these effects. These findings highlight the importance of the interactions between signalling pathways and transcriptional programs that drive endocrine cell differentiation.

## Results

### Neurog3 induces the *Wnt9a* gene *in vitro*

The mouse mPAC L20 cell line has been previously used to model Neurog3-dependent induction of the endocrine differentiation program^21^. Using gene expression arrays^13^, we identified several Wnt-related genes including ligands, receptors, modulators, effectors and targets, whose expression was regulated by Neurog3 in this model (Supplementary Table S1). These findings led us to investigate the involvement of Wnt signals in endocrine cell development.

**Figure 1:**
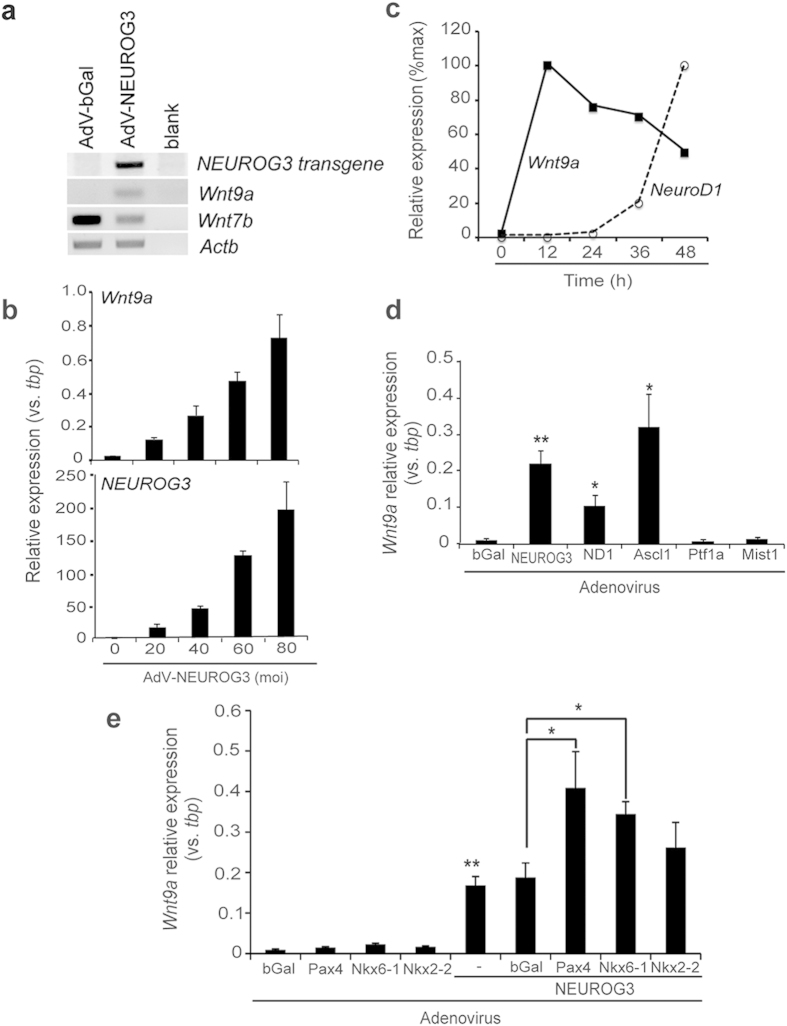
Regulation of the *Wnt9a* gene by NEUROG3 *in vitro*. mPAC cells were treated with the indicated adenoviruses and total cellular RNA was extracted 48 h after virus treatment. (**a**) mRNAs encoding the indicated genes were assessed by conventional RT-PCR. Beta actin (*Actb*) was used as internal control. Cycles used were 25 for *Actb* and 28 for the other genes. Representative gel image is shown. (**b**) mPAC cells were treated with the indicated moi of AdV-NEUROG3. *Wnt9a* and *NEUROG3*-transgene expression were measured by qRT-PCR and expressed relative to TATA-binding protein (*tbp*) gene expression. Bars represent mean ± SEM for at least 3 independent experiments. (**c**) mPAC cells were treated with AdV-NEUROG3 (moi = 40) and collected at the indicated time points after addition of the virus. *Wnt9a* mRNA levels were quantitated by qRT-PCR. RNA levels for *NeuroD1*, as a control for a late Neurog3 target, were also assayed. Expression was normalized with *tbp*. To facilitate comparisons, for each gene the time point with highest expression was assigned a value of 100, and other time points expressed relative to this one. Symbols represent mean for 2 independent experiments. Note that *Wnt9a* mRNA reaches highest levels 12h after virus treatment. (**d**,**e**) *Wnt9a* mRNA levels were quantitated by qRT-PCR and expressed relative to *tbp* gene expression. Bars represent mean ± SEM for at least 3 independent determinations. *p < 0.05, **p < 0.01 vs. AdV-bGal.

Two ligands emerged as potential Neurog3 targets in our microarray data set, Wnt9a and Wnt7b (Table S1 and Fig. 1a). *Wnt7b* has been recently ascribed a role in pancreatic progenitor proliferation before the secondary transition^10^, hence we focused on *Wnt9a* as a potential novel Neurog3 target. We conducted time and dose curves for *Wnt9a* induction and confirmed early activation of this gene upon Neurog3 expression (Fig. 1b,c). To assess the specificity of the Neurog3 actions, we investigated the effects of other pancreatic and neuronal lineage-promoting bHLH factors on *Wnt9a* mRNA levels. We found that NeuroD1 (neuroendocrine) and Ascl1 (neural) activated *Wnt9a* gene expression at similar levels as Neurog3, whereas the exocrine bHLH factors Ptf1a and Mist1 failed to do so (Fig. 1d), placing the *Wnt9a* gene downstream of lineage-driving neuroendocrine bHLH factors. To further delineate the position of Wnt9a in the endocrine program, we assessed the effects of the endocrine differentiation factors Pax4, Nkx6-1 and Nkx2-2, which are upregulated downstream of Neurog3. When expressed alone, they had no effect, but Pax4 and Nkx6-1 potentiated Neurog3-dependent transactivation of the *Wnt9a* gene (Fig. 1e). Neurog3 also activated the *Wnt9a* gene in non-pancreatic cell contexts such as teratocarcinoma P19 cells and 3T3 fibroblasts (Supplementary Fig. S1), thus supporting the sufficiency of Neurog3 to increase *Wnt9a* expression *in vitro.*

### *Wnt9a* is expressed in the embryonic pancreas

**Figure 2:**
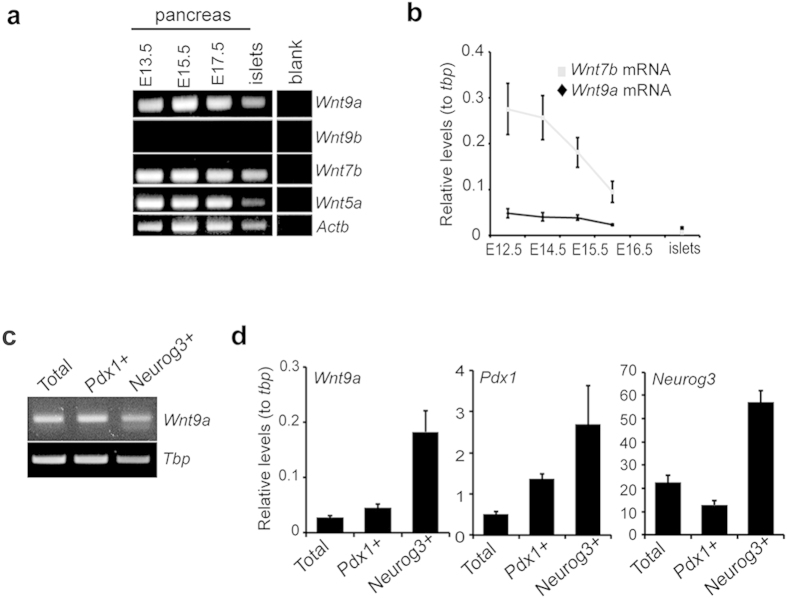
Expression of *Wnt9a* in the embryonic pancreas. (**a**) Total RNA was extracted from mouse embryonic pancreases at the indicated stages and from adult isolated mouse islets. mRNAs encoding the indicated genes were assayed by conventional RT-PCR. Note that expression of both *Wnt5a* and *Wnt7b* had been previously identified in the developing pancreas and are included for comparison purposes. Cycles used were 25 for *Actb* and 35 for the other genes. Representative gel image is shown. Lane showing non-template control for each transcript (blank) was run under the same experimental conditions. (**b**) *Wnt9a* and *Wnt7b* mRNA levels were quantitated by qRT-PCR and expressed relative to *tbp* gene expression. Each point represents mean ± SEM for at least 3 independent determinations. (**c**) Representative gel showing expression of *Wnt9a* mRNA in E15.5 total pancreas, in FACS-purified Tomato+ (Pdx1+, epithelial) cells obtained from E14.5 pancreas of *Pdx1*-Cre; Rosa26: tdTomato and in FACS-purified Tomato+ (Neurog3+, endocrine) cells obtained from E15.5 pancreas of *Neurog3*-Cre; Rosa26: tdTomato. (**d**) Quantification of *Wnt9a, Neurog3* and *Pdx1* mRNA levels in total RNA from samples prepared as detailed in (**c**). Values are expressed relative to *tbp* mRNA levels in each sample. Bars represent mean ± SEM for at least 5 independent sorting experiments.

Prior to investigating the role of Wnt9a during endocrine cell development *in vivo*, we examined the presence of *Wnt9a* transcripts in the developing mouse pancreas. We performed conventional RT-PCR using RNA from E13.5-E17.5 pancreatic buds and confirmed that *Wnt9a* was expressed in the pancreas during the secondary transition (Fig. 2a). To assess its kinetics of expression, we used qRT-PCR and found that the *Wnt9a* message was expressed at low and relatively constant levels, in sharp contrast to *Wnt7b*, throughout the stages studied (Fig. 2b).

In lieu of a suitable antibody we studied lacZ activity as readout for *Wnt9a* expression in pancreases from *Wnt9a*^*L*acZ^ mice^16^. Although we were unable to detect a reproducible signal in heterozygous Wnt9a^*LacZ/+*^ pancreases (likely due to low expression levels), we did observe positive signal in central areas of the pancreatic epithelium in knockout *Wnt9a*^*L*acZ/*LacZ*^ pancreases at E14.5-E15.5 after lengthy X-gal staining (Fig. S2). To obtain additional evidence for the expression of the *Wnt9a* gene in pancreatic epithelial cells, we obtained E14.5 pancreases from *Pdx1*-Cre; Rosa26:tdTomato (tdT) mice, isolated tdT+ cells by FACS and verified the presence of *Wnt9a* transcripts in Pdx1+ cells (Fig. 2c,d). Of note, *Wnt9a* mRNA was also detected in Pdx1- cells (data not shown), indicating that this gene is also expressed in the pancreatic mesenchyme as described for other Wnt ligands^3^. We next isolated tdT+ cells from *Neurog3*-Cre; Rosa26:tdT pancreases at E15.5 and established expression of *Wnt9a* in endocrine progenitor cells and their descendants (Fig. 2c,d). Altogether, these observations place Wnt9a at the right time and place to play an autonomous role in pancreatic endocrine cell differentiation.

### *Wnt9a* ablation leads to increased pancreatic endocrine cell numbers

**Figure 3:**
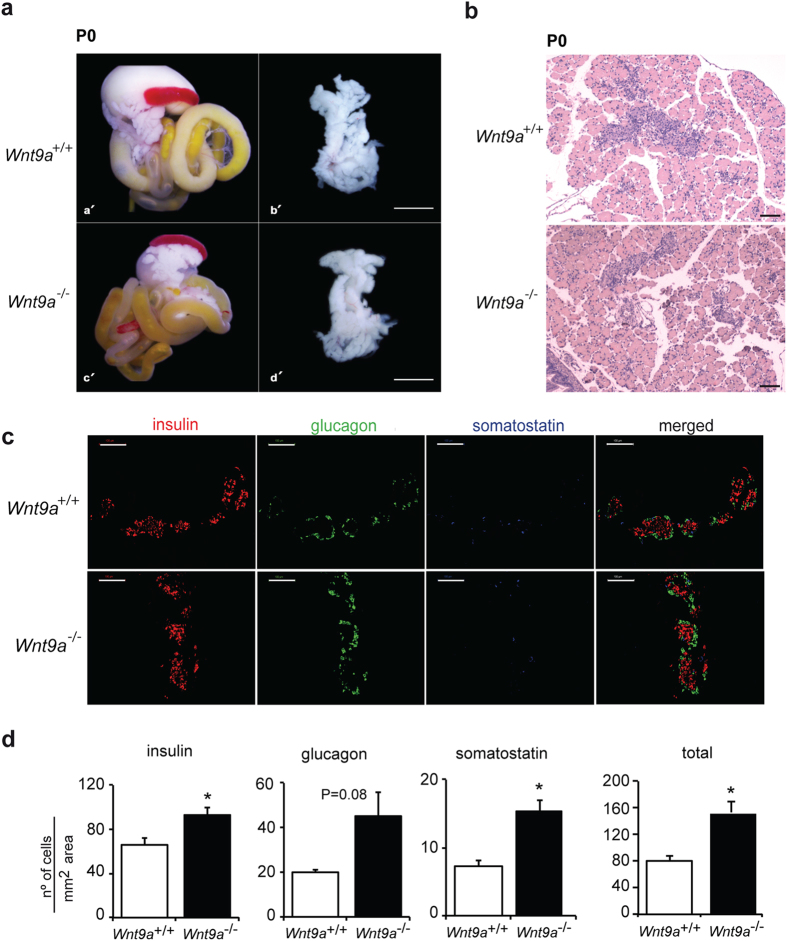
Pancreatic phenotype of *Wnt9a* knockout embryos. (**a**) Examination of gross morphology of digestive tract (a’,c’) and pancreas (b’,d’) from neonatal *Wnt9a*^+/+^ (a’,b’) and *Wnt9a*^−/−^ (c’,d’) mice. Scale bar is 5000 μm. (**b**) Hematoxylin and Eosin staining was performed on paraffin sections from pancreatic tissue from neonatal *Wnt9a*^+/+^ and *Wnt9a*^−/−^ mice. Scale bar is 200 μm. (**c**) Staining for insulin (red), glucagon (green) and somatostatin (blue) on paraffin sections from pancreases of E18.5 *Wnt9a*^+/+^ and *Wnt9a*^−/−^ embryos. Scale bars represent 100 μm. (**d**) Morphometric quantification of the number of endocrine cells per pancreatic area in E18.5 *Wnt9a*^+/+^ and *Wnt9a*^−/−^ embryos. Positive cells for each indicated hormone were counted separately and for the number of total endocrine cells combined (total). Bars represent mean ± SEM from 5 animals per genotype. *p < 0.05 vs Wnt9a^+/+^.

To address the function of Wnt9a during endocrine pancreatic development, we undertook a loss-of-function approach and characterized the pancreatic phenotype of *Wnt9a* knockout mice generated by crossing *Wnt9a*^*LacZ/+*^ mice. Homozygous *Wnt9a*^*LacZ/LacZ*^ (hereafter *Wnt9a*^−/−^) pups fail to thrive and die within the first hours of birth of undefined causes^16^. Pancreas weight, in absolute values (WT: 8.40 ±0.59mg; *Wnt9a*^−/−^: 7.83 ±0.82 mg) and as percentage of body weight (WT: 7.61 ±0.52; *Wnt9a*^−/−^: 6.90 ±0.2), was similar between *Wnt9a*^−/−^ and wild-type (WT) newborn littermates. Similarly, there were no observable differences in pancreas gross morphology and architecture (Fig. 3a,b), demonstrating that Wnt9a is not necessary for pancreas formation and growth. Next we stained pancreas sections from E18.5 embryos with antibodies against insulin (β-cells), glucagon (α-cells) and somatostatin (δ-cells) confirming the presence and normal spatial cell disposition of these islet lineages in *Wnt9a*^−/−^ pancreases (Fig. 3c). We performed morphometric analysis to evaluate the relative abundance of these cell types and found that β-cells were increased by 1.4-fold, α-cells by 2.2-fold and δ-cells by 2.1-fold (pancreatic areas analyzed: 11.12 ± 1.27 mm^2^ for *Wnt9a*^−/−^ and 11.45 ± 1.28 mm^2^ for WT) in *Wnt9a*^−/−^ pancreases relative to WT (Fig. 3d), thus revealing that *Wnt9a* loss leads to higher endocrine cell numbers. Unfortunately, early postnatal lethality of *Wnt9a* knockouts prevented assessment of the impact of this phenotype on glucose homeostasis.

As Wnt signaling controls β-cell proliferation^22^, we assessed whether enhanced proliferation contributed to increased β-cell counts at E18.5. At E17.5, the total endocrine area (measured as chromogranin A+) was higher in *Wnt9a*^−/−^ embryos (Supplementary Fig. S3), but there were no differences in the percentage of double positive Ki67/insulin cells between *Wnt9a*^−/−^ and WT pancreases (Supplementary Fig. S3). Therefore, increased proliferation does not appear to contribute to increased β-cell numbers in *Wnt9a*-deficient embryos, pointing to a role of Wnt9a-dependent signalling in endocrine cell genesis.

### Wnt9a deficiency upregulates endocrine gene expression in the embryonic pancreas

**Figure 4:**
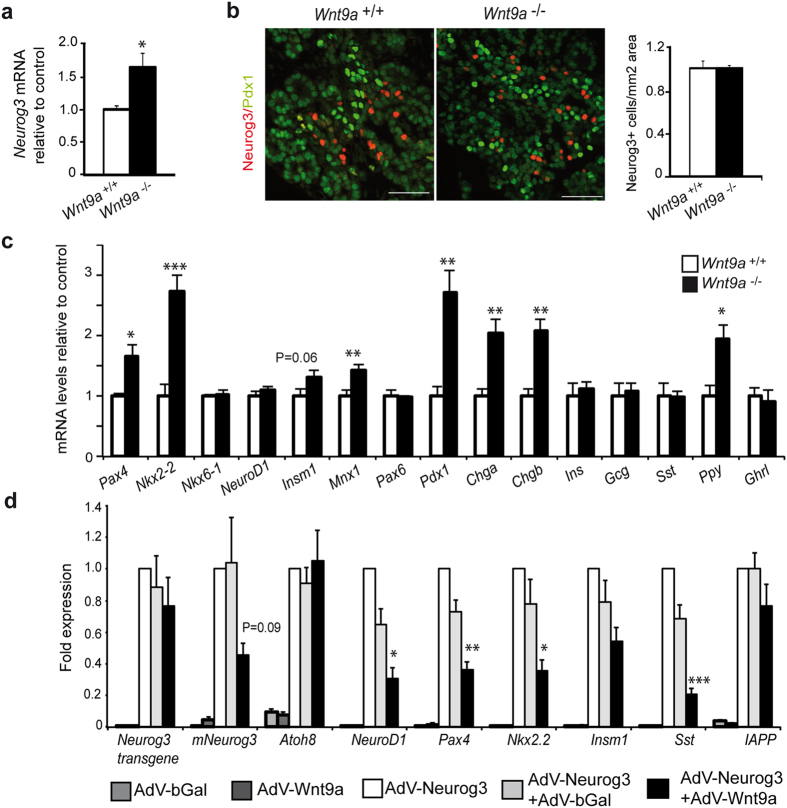
Effects of *Wnt9a* ablation on activation of the endocrine gene expression program. (**a**) Total RNA was isolated from pancreases of E15.5 *Wnt9a*^+/+^ and *Wnt9a*^−/−^ embryos. *Neurog3* mRNA expression was assessed by qRT-PCR and normalized against *tbp*. Results are expressed as fold relative to levels in *Wnt9a*^+/+^ pancreases (value of 1). Bars represent mean ± SEM for 8 *Wnt9a*^+/+^ and 7 *Wnt9a*^−/−^ embryos. *p < 0.05 vs *Wnt9a*^+/+^. (**b**) Double immunostaining for Neurog3 (red) and Pdx1 (green) on paraffin sections from pancreases of E15.5 *Wnt9a*^+/+^ and *Wnt9a*^−/−^ embryos. Scale bars represent 50 μm. Quantification of the number of Neurog3 + cells per pancreatic area is shown on the right. Bars represent mean ± SEM for 3 animals per genotype. (**c**) Total RNA was isolated from pancreases of E15.5 *Wnt9a*^+/+^ and *Wnt9a*^−/−^ embryos. mRNA levels for the indicated genes were quantitated by qRT-PCR and normalized against *tbp*. Results are expressed as fold relative to levels in *Wnt9a*^+/+^ pancreases (value of 1). Bars represent mean ± SEM for 8 *Wnt9a*^+/+^ and 7 *Wnt9a*^−/−^ embryos. *p < 0.05, **p < 0.01 vs *Wnt9a*^+/+^. (**d**) mPAC cells were transduced with the indicated adenoviruses alone or in combination. Total cellular RNA was isolated 48 h after virus treatment. mRNAs encoding the indicated genes were assessed by qRT-PCR and normalized relative to *tbp*. Expression in cells treated with AdV-Neurog3 was given a value of 1. Bars represent mean ± SEM for 6–8 independent experiments. *p < 0.05, **p < 0.01, ***p < 0.001 vs AdV-Neurog3+ AdV-bGal.

During development, islet cells differentiate from bipotent duct/endocrine progenitors located in the trunk domain of the growing pancreas. To establish whether the increase in endocrine cells observed in E18.5 *Wnt9a*^−/−^ embryos resulted from augmented endocrine allocation from progenitors, we determined *Neurog3* transcript levels and counted Neurog3+ cells by immunofluorescence at E15.5, the peak of the secondary transition. We found a modest increase in *Neurog3* mRNA levels (Fig. 4a) whereas the number of Neurog3+ cells was unchanged (Fig. 4b), suggesting that *Wnt9a* loss does not affect endocrine specification but it may influence *Neurog3* levels per cell. Yet, we did not detect obvious discrepancies in the relative proportion of high and low Neurog3+ expressing cells between mutants and controls (Fig. 4a and data not shown), thus indicating that modest increase in *Neurog3* mRNA detected upon *Wnt9a* loss most likely results in undetectable changes in Neurog3 protein amount, at least by conventional immunofluorescence.

We then used qRT-PCR to screen a panel of endocrine genes activated downstream of Neurog3. Remarkably, we found that mRNA levels for several of these genes, namely *Nkx2-2* and *Pdx1* and to a lesser extent *Pax4* and *Mnx1* were increased in E15.5 *Wnt9a*^−/−^ pancreases relative to WT (Fig. 4c). Also, message levels for the early pan-endocrine markers *Chromogranin a* and *b* (*Chga, Chgb*) and the islet hormone *Pancreatic Polypeptide* (*Ppy*) were higher in *Wnt9a*^−/−^ relative to WT (Fig. 4c). Therefore, *Wnt9a* ablation results in the upregulation of a subset of genes in the endocrine differentiation program downstream of Neurog3.

To test if Wnt9a could directly impair the pro-endocrine activity of Neurog3, we generated a recombinant adenovirus encoding Wnt9a (AdV-Wnt9a) and ectopically expressed this ligand, alone or in combination with Neurog3, in mPAC cells. While Wnt9a alone had no effects on any of the genes studied (not endogenously expressed in mPAC cells except for *Atoh8*), it partially blocked activation of *NeuroD1, Pax4, Nkx2-2* and *Sst* in response to Neurog3 (Fig. 4d). Remarkably, Wnt9a tended to decrease Neurog3 positive autoregulation^23^, which agrees with the finding that *Neurog3* transcripts are increased in *Wnt9a* mutants. By contrast, Wnt9a did not affect Neurog3-dependent activation of *Atoh8, Insm1* and *IAPP*. In summary, the *in vivo* and *in vitro* results support a negative role of Wnt9a on endocrine differentiation.

To assess the specificity of the effects of *Wnt9a* in the endocrine compartment, we studied expression of acinar and ductal genes at E15.5. We found no changes in early (*Cpa1, Ptf1a, Mist1*) or mature (*amylase*) acinar cell markers, thus excluding a major effect of Wnt9a in the acinar program (Supplementary Fig. S4a). In contrast, mRNA levels for several ductal markers (*Muc1, Krt19, Spp1, Pkd2, Hnf1b*) were significantly reduced (Supplementary Fig. S4a). This effect did not seem to be dependent on Sox9, a ductal fate determinant^24^, as no differences in either its expression or immunolocalization (Supplementary Fig. S4a,b) were observed between WT and *Wnt9a* knockout embryos. Despite decreased gene expression at E15.5, ductal differentiation appears to proceed normally in *Wnt9a*^−/−^ embryos as indicated by normal appearance and quantification of the ductal tree at E17.5 using DBA staining (Supplementary Fig. S4c). A transitory embryonic ductal phenotype that resolves at later stages has also been described in *Neurog3* mutants^25^.

### *Wnt9a*-deficient embryos present diminished *Tcf7l2* gene expression

**Figure 5:**
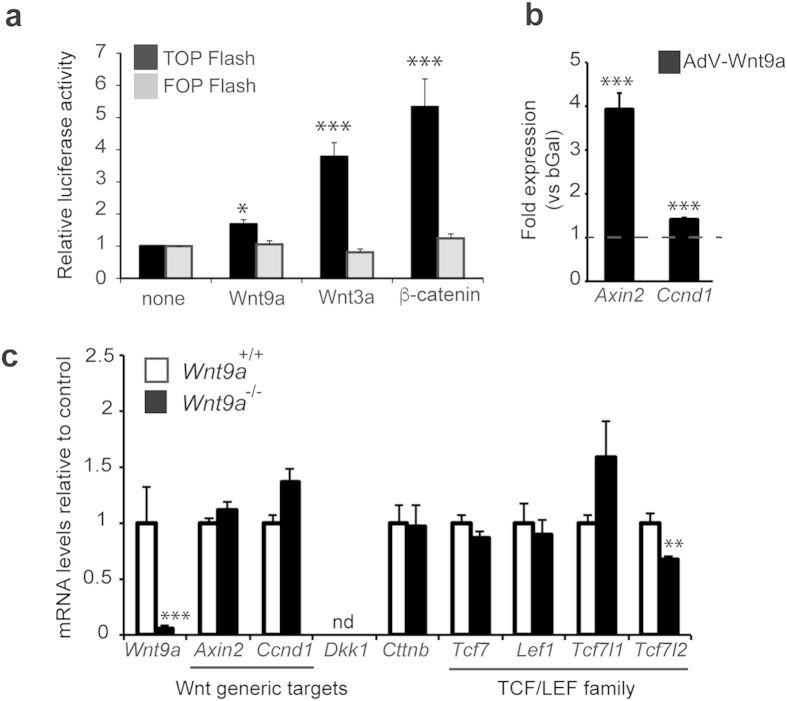
Involvement of TCF/LEF factors in Wnt9a-dependent signalling. (**a**) mPAC cells were transfected with the TCF-dependent luciferase reporter vector TOP-Flash (black) or its mutated version FOP-Flash (grey) and expression vectors for Wnt9a, Wnt3a or a mutant form of Ctnnb1 lacking aminoacids 29–48. Results are expressed relative to the backbone vector, normalized to 1. Bars represent mean ± SEM for at least 4 independent experiments, each performed in duplicate *p < 0.05 and ***p < 0.001 vs backbone. (**b**) mPAC cells were transduced with an adenovirus expressing Wnt9a (AdV-Wnt9a) or AdCMV-bGal as control and total cellular RNA was isolated 48 h after virus treatment. mRNA levels for *Axin2* and *Ccnd1* were quantitated by qRT-PCR and normalized to *tbp*. Values are expressed relative to cells treated with AdV-bGal, which are given the value of 1. Bars represent mean ± SEM for at least 7 independent experiments. ***p < 0.001 vs bGal. (**c**) Total pancreatic mRNA from *Wnt9a*^+/+^ and *Wnt9a*^−/−^ animals was collected at E15.5 and expression for the indicated genes was assessed by qRT-PCR and normalized with *tbp*. Results are presented relative to Wnt9a^+/+^ given the value of 1. Bars represent mean ± SEM for 7 *Wnt9a*^+/+^ and 6 *Wnt9a*^−/−^ animals. *p < 0.05 vs *Wnt9a*^+/+^. Note that *Dkk1* mRNA was not detected in either knockout or wild-type pancreas (ND = not detected).

Wnt9a has been regarded as a canonical Wnt ligand^14^,^16^,^20^. In initial set-up experiments using a Tcf-dependent luciferase reporter vector, we surveyed several pancreatic cell lines (α, β and ductal) and found that basal pathway activity and responsiveness to β-catenin overexpression was very low, being mPAC cells the ones that provided the best response (data not shown). Hence, we used these cells to interrogate whether Wnt9a signalling was transduced via Tcf factors in a pancreatic context. In accordance to a canonical role, Wnt9a increased the activity of the Tcf-dependent reporter vector and augmented endogenous mRNA levels of the *bona fide* Wnt targets (known to be transcriptionally induced by the β-catenin/Tcf pathway) *axin2* and *Ccnd1* in mPAC cells (Fig. 5a,b). We then tested whether *Wnt9a* ablation affected expression of these general Wnt targets *in vivo* using RNA from total pancreas but found no differences between knockout and WT embryos (Fig. 5c). Whilst these results are compatible with Wnt9a signalling affecting a restricted cell population in the pancreas, they expose that Wnt9a is not a principal regulator of canonical Wnt activity in the pancreas at E15.5. In fact, expression of several Wnt ligands has been demonstrated in the pancreas at this stage, which could have redundant effects on maintenance of general Wnt target expression^3^,^10^.

In addition to the above-mentioned classical Wnt targets, Wnt signalling regulates expression of genes in a cell context-specific manner. Among the latter, genes encoding LEF/TCF proteins, the effectors of the pathway, have been shown to be subject to auto-regulation in several developmental contexts^26^,^27^,^28^. Hence, we tested whether *Wnt9a* loss influenced pancreatic expression of the LEF/TCF coding genes (*Tcf7, Lef1, Tcf7l1, Tcf7l2*). We found that all four genes were expressed in the pancreas at E15.5. Remarkably, we observed a significant and specific reduction in the expression of *Tcf7l2* in *Wnt9a*^−/−^ pancreases relative to controls, while the other LEF/TCF and β-catenin (*ctnnb*) coding genes were similarly expressed in WT and knockout pancreases (Fig. 5c). Collectively, these data disclose Tcf7l2 as a potential target/mediator of Wnt9a signalling in the developing pancreas.

### Tcf7l2 negatively regulates endocrine gene expression *in vitro*

**Figure 6:**
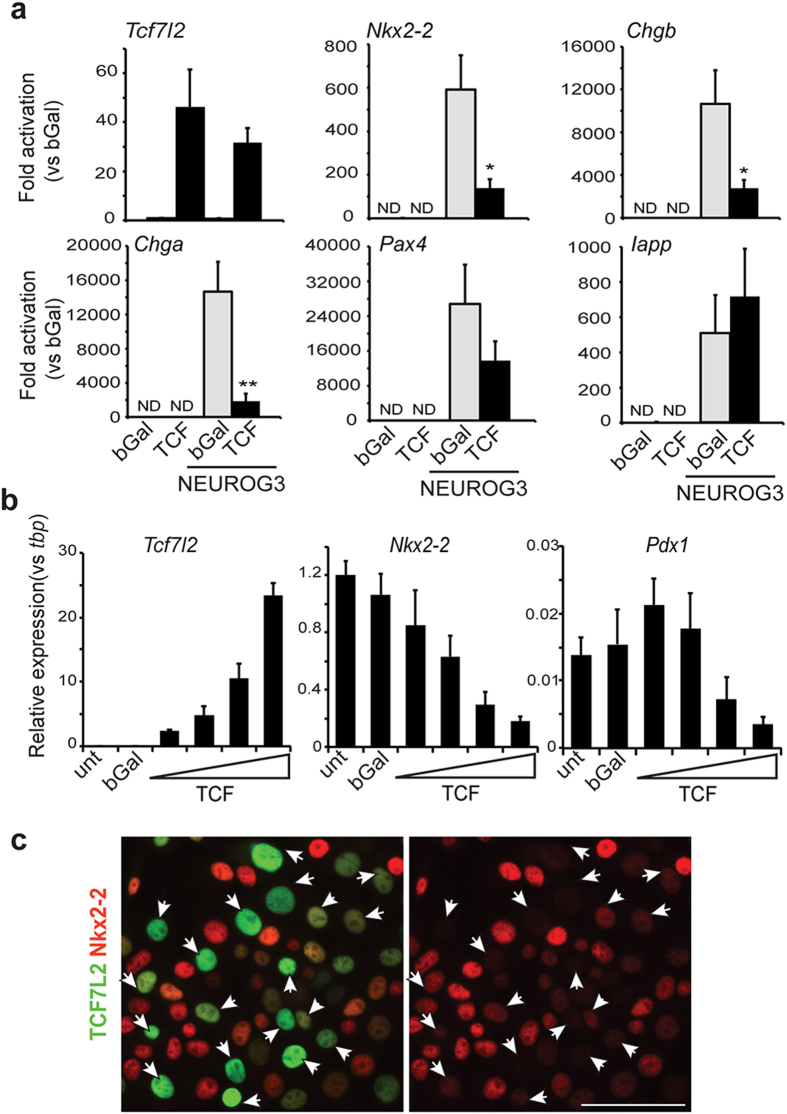
Regulation of the *Nkx2-2* gene by Tcf7l2. (**a**) mPAC cells were treated with the indicated adenoviruses (bGal, Neurog3 or TCF7L2) and total cellular RNA and/or total protein extracts were prepared 48 h after virus treatment. mRNA levels for the indicated genes were measured by qRT-PCR and expressed relative to *tbp*. Data is expressed relative to levels in cells treated with AdV-bGal. Bars represent mean ± SEM for 4 independent experiments. ND = not detectable (Ct > 36). *p < 0.05 **p < 0.01 vs Bgal. (**b**) INS1E cells were treated with increasing amounts of the adenovirus encoding TCF7L2 and total cellular RNA was extracted 24 h after virus treatment. Gene expression for *Tcf7l2* (primers amplify both endogenous and adenovirally-expressed gene), *Nkx2-2* and *Pdx1* were measured by qRT-PCR and expressed relative to *tbp* gene expression. Bars represent mean ± SEM for 3-4 independent experiments. (**c**) Double immunofluorescence staining for TCF7L2 (green) and Nkx2-2 (red) in INS1 cells 24 h after transduction with an adenovirus encoding human TCF7L2. Arrows point to cells expressing adenovirally-expressed TCF7L2. Note that endogenous Tcf7l2 levels were undetectable.

We next interrogated the existence of a mechanistic connection between Tcf7l2 and endocrine differentiation. Interestingly, Tcf7l2 had been shown to repress the *Nkx2-2* gene in the developing neural system^29^. This evidence together with our findings that *Wnt9a*-deficient pancreases present enhanced expression of *Nkx2-2* and of some of its downstream targets (namely *Pdx1, Mnx1, Chga, Chgb* and *Ppy*^30^,^31^,^32^) led us to postulate that Tcf7l2 might negatively regulate *Nkx2-2* in the developing pancreas. To examine this possibility, we studied *Nkx2-2* gene activation in response to Neurog3 in the absence or presence of Tcf7l2 in mPAC cells. Overexpressed Tcf7l2 blocked by nearly 75% Neurog3-induced activation of the *Nkx2-2* gene (Fig. 6a). Similar results were obtained using a dominant-negative version of Tcf7l2 that lacked the N-terminal β-catenin binding domain (data not shown), pointing to the involvement of the repressor function of Tcf7l2, not its binding to β-catenin. Moreover, overexpressed Tcf7l2 reduced Neurog3-triggered induction of additional Neurog3 targets including *Chga* and *Chgb* (Fig. 6a).

We also asked if Tcf7l2 could inhibit *Nkx2-2* gene expression in differentiated β-cells where the *Nkx2-2* gene is actively expressed. Indeed, Tcf7l2 reduced *Nkx2-2* mRNA and protein levels in the β-cell line INS1E (Fig. 6b,c). Overexpressed Tcf7l2 also downregulated *Pdx1* transcript levels (note that Pdx1 was also upregulated in *Wnt9a*-ablated pancreases) in INS1E cells (Fig. 6b). Together, these results reveal a negative effect of Tcf7l2 on the transactivation activity of Neurog3 and provide support to the notion that Tcf7l2 can negatively affect endocrine differentiation.

### Tcf7l2 expression is downregulated in the differentiating endocrine compartment *in vivo*

In order to understand if Tcf7l2 could regulate *Nkx2-2* and endocrine differentiation *in vivo*, we characterized the expression pattern of Tcf7l2 in the pancreas during the secondary transition. To obtain spatial information on Tcf7l2 expression, we used two antibodies: C9B9 raised against an epitope downstream of the β-catenin binding domain (Glu81 of human TCF7L2) and C48H11 raised against an epitope close to the HMG-box DNA binding domain (Leu330 of human TFC7L2). Both antibodies recognize long E (∼80KDa) and short M and S (∼58KDa) isoforms^33^, whilst C48H11 also recognizes a recently identified short truncated isoform (35-37KDa) that lacks the β-catenin binding domain and functions as a dominant negative Wnt antagonist^34^. We detected the presence of the two major groups of long and short isoforms but not the dominant negative protein in total protein extracts from E15.5 pancreases by immunoblot analysis (Supplementary Fig. S5a). When tested for immunostaining, only C48H11 provided suitable signal and revealed broad expression of Tcf7l2 throughout the pancreatic epithelium, marked by Pdx1 expression, at E15.5 (Supplementary Fig. S5b). As expected from the 30% reduction seen at the mRNA level, no readily observable differences were noted in overall Tcf7l2 expression between *Wnt9a*^−/−^ and control pancreases using immunostaining (Supplementary Fig. S6).

**Figure 7:**
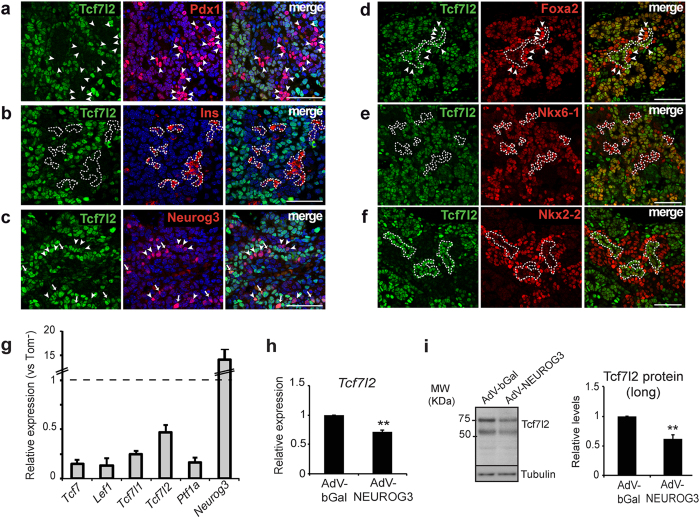
Expression patterns of Tcf7l2 in the embryonic pancreas during the secondary transition. (**a–f**) Co-immunostaining of Tcf7l2 (green) with: (**a**) Pdx1 (red); (**b**) insulin (red); (**c**) Neurog3 (red); (**d**) Foxa2 (red); (**e**) Nkx6-1 (red) and (**f**) Nkx2-2 (red); on paraffin sections of pancreas from *Wnt9a*^+/+^ and *Wnt9a*^−/−^ embryos at E15.5. Arrowheads depict cells expressing Pdx1 (**a**), Neurog3 (**c**) or Foxa2 (**d**) and very low or undetectable Tcf7l2. Arrows in c depict cells where Neurog3 and Tcf7l2 appear to be co-expressed. Scale bars are 50 μm. (**g**) mRNA levels for the indicated genes were quantitated in FACS-purified Tomato + (endocrine) cells obtained from E15.5 pancreas of *Neurog3*-Cre; Rosa26: tdTomato. Results are expressed relative to values in Tomato- cells isolated in the same experiments (value of 1). Bars represent mean ± SEM for 5 independent sorting experiments. Transcript levels for *Ptf1a* and *Neurog3* are shown to illustrate non-endocrine vs. endocrine marker enrichment of Tomato + cells. (**h-i**) mPAC cells were treated with the indicated adenoviruses (bGal or Neurog3) and total cellular RNA and total protein extracts were prepared 48 h after virus treatment. (**h**) *Tcf7l2* mRNA levels were measured by qRT-PCR and expressed relative to *tbp* gene expression. Bars represent mean ± SEM for 8 independent experiments. (**i**) Immunoblot analysis of Tcf7l2 protein levels. Densitometric values were normalized to Tubulin and expressed relative to cells transduced with AdV-Bgal, which were given the value of 1. Bars represent mean ± SEM for 6 independent experiments. **p < 0.01 vs Bgal.

However, we identified areas with very low or nearly undetectable Tcf7l2 staining both in control and mutant pancreases. These cells displayed high Pdx1 expression (Fig. 7a) and were insulin-positive (Fig. 7b), indicating that differentiating or young β-cells contain little or no Tcf7l2 protein, at least some isoform/s recognized by the C48H11 antibody. We also found that most Neurog3+ cells displayed undetectable or very low Tcf7l2 levels, except for a few cells where co-expression was evident (Fig. 7c), suggesting a transition between expression of these proteins. Marginal Tcf7l2 expression was also observed in cells expressing high levels of Foxa2 located within or adjacent to Tcf7l2 positive epithelial chords, which correspond to cells at early stages of endocrine differentiation^23^ (Fig. 7d). Likewise, Tcf7l2 was co-expressed with Nkx6-1 in epithelial chords but was low in some cells exhibiting high Nkx6-1 levels, which likely correspond to differentiating β-cells (Fig. 7e). Remarkably, robust Tcf7l2 signal was detected in Nkx2-2-low trunk progenitors. Conversely, cells expressing Nkx2-2 (differentiating endocrine compartment) exhibited low or absent Tcf7l2 expression (Fig. 7f), revealing a well-defined mutually exclusive pattern of expression of these two transcription factors that would be in agreement with negative regulation of Nkx2-2 by Tcf7l2.

Lastly, to confirm the immunostaining data, we determined *Tcf7l2* mRNA levels in Neurog3 + progenitors and their descendants using FACS-purified tdT+ cells from Neurog3-Cre;tdT embryos. *Tcf7l2* was expressed in tdT+ cells but its levels were reduced by 53% relative to tdT- (non-endocrine) cells. Strikingly, all LEF/TCF genes appeared to be downregulated in endocrine cells relative to the non-endocrine compartment (Fig. 7g). Since most Neurog3+ cells had very low or undetectable Tcf7l2 protein levels (Fig. 7c), we reasoned that Tcf7l2 expression decayed at the time of or shortly after endocrine specification. Interestingly, we observed that forced expression of Neurog3 reduced mRNA and protein levels of Tcf7l2 in mPAC cells (Fig. 7h,i), thus supporting a direct connection between activation of endocrine differentiation and downregulation of Tcf7l2 expression.

In sum, these findings reveal that Tcf7l2 levels decline in endocrine-committed progenitors and in the differentiating endocrine compartment relative to cells within the epithelial progenitor chords at E15.5. Therefore, reduced *Tcf7l2* gene expression is compatible with enhanced endocrine differentiation in *Wnt9a*-ablated pancreases.

## Discussion

The function of the Wnt signalling pathway is highly dependent on cell context, and this dependency relies in part on its ligands and receptors, which display high lineage-specificity and are the most dynamically regulated components of the pathway during development^35^. However, many of the studies aimed at investigating the role of Wnt signalling in pancreas formation have used genetic manipulation of common core machinery, namely β-catenin, which may have masked more subtle cell type-specific and stage-dependent effects of this pathway. In the current study we aimed at defining the involvement of Wnt signals in endocrine cell formation through the investigation of the ligand Wnt9a, whose gene we identified as a target of the pro-endocrine transcription factor Neurog3 in cultured cells. Interestingly, publicly available gene expression profiling data shows that *Wnt9a* expression is higher in Neurog3+ than in Sox9+ (bipotent duct/endocrine progenitors) cells at E15.5 (GEO ID = GDS4335: 10376490), consistent with this ligand being activated downstream of Neurog3. Whether Neurog3 regulates the *Wnt9a* gene directly or indirectly remains to be determined. It is noteworthy that both *Wnt9a and* Wnt7b (also identified as a Neurog3 target in our microarray analysis) were predicted as potential direct transcriptional targets of Neurogenin/NeuroD factors during neurogenesis using an informatics-based screening approach^36^.

**Figure 8:**
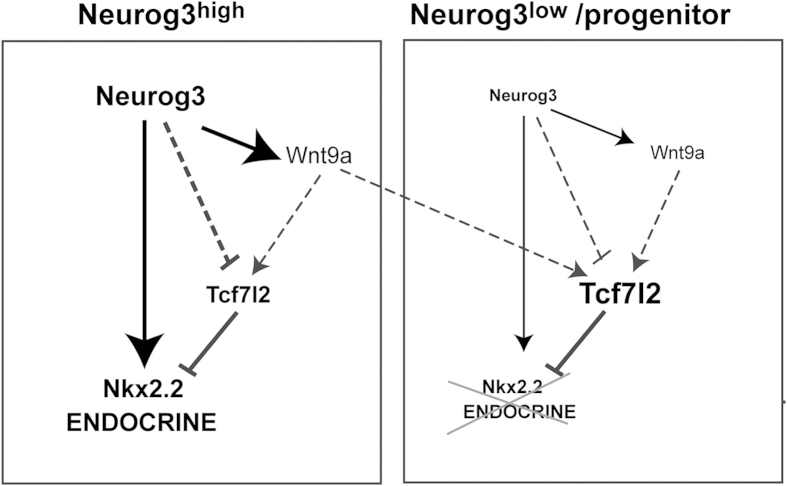
Proposed model for regulation of endocrine differentiation by Wnt9a. The proposed model is based on (1) Neurog3 upregulates expression of the *Nkx2-2* and *Wnt9a* genes and (2) Tcf7l2 negatively regulates the *Nkx2-2* gene. Hence, in Neurog3+ cells, Wnt9a signalling would increase Tcf7l2 activity thus reducing Neurog3-dependent induction of the *Nkx2-2* gene and serving as a control brake for activation of the endocrine program. In Wnt9a knockout animals, loss of Wnt9a would lead to reduced Tcf7l2 and enhanced *Nkx2-2* expression. Additionally, Wnt9a may exert a paracrine effect and reinforce alternative non-endocrine fates by maintaining high Tcf7l2 expression/activity in neighbor cells. Note that Neurog3 may also have a direct repressive role on Tcf7l2 expression, which would ultimately help reinforce activation of the endocrine program in cells expressing sufficient levels of this pro-endocrine factor. Dashed lines indicate regulatory links where precise molecular mechanisms remain to be defined.

The phenotype of *Wnt9a* mutants exposes a negative effect of this ligand in endocrine cell genesis, thus suggesting that Wnt9a functions in a negative feedback loop to limit Neurog3 activity *in vivo*. Indeed, the absence of changes in pancreas growth, endocrine cell specification or endocrine cell proliferation in *Wnt9a* mutants is compatible with a role of Wnt9a during activation of the endocrine program downstream of Neurog3. Based on the findings presented here, we propose a model whereby levels of the Wnt effector Tcf7l2 need to be down regulated in endocrine progenitors to permit effective activation of the endocrine program. In this scenario, Wnt9a-dependent signalling, via positive regulation of Tcf7l2 expression/activity, would work as a control brake for endocrine differentiation (Fig. 8). Both *in vivo* and *in vitro* evidences point to Nkx2-2, whose requirement for correct endocrine cell development has been long established^37^, as an important target of the Wnt9a-Tcf7l2 pathway in the embryonic pancreas, although additional targets cannot be ruled out. If we assume that this model is correct and consider that not all cells that turn on Neurog3 expression ultimately become endocrine cells^38^, it can be speculated that under the *Wnt9a*-null condition, endocrine differentiation would be facilitated in cells that would otherwise revert to alternate (exocrine) fates, i.e cells that turn on a low level of Neurog3^38^. The down-regulation of ductal gene expression in *Wnt9a*- ablated pancreases further supports the contribution of this ligand to fine-tuning activation of endocrine versus ductal cell programs in the pancreas.

Prior studies have demonstrated a role of another signalling pathway, the Notch pathway, in the regulation of the endocrine/ductal binary fate decision^24^. Interestingly, we have found that Notch ligand genes are upregulated in *Wnt9a*-ablated pancreases (GP, RG unpublished observations), which suggests that Notch may be involved in the effects of Wnt9a in the pancreas. The interplay Wnt-Notch is often seen in development^39^ and Notch ligands have been identified as Wnt targets in several contexts^40^,^41^,^42^,^43^. Since Neurog3 regulates Notch ligand gene expression^21^,^44^,^45^, it appears that a complex network of cross-regulatory interactions between Neurog3 and these signalling pathways controls endocrine cell fate acquisition in the pancreas. Future work is needed to determine the molecular underpinnings of this network at a cell-based resolution in models with single and combined tissue-specific manipulations.

Genetic variations of the gene coding for TCF7L2 have been associated with type 2 diabetes in humans^46^. Remarkably, the *TCF7L2* variant conferring the strongest risk for diabetes is suspected to exert its effects through increasing transcriptional activity of the *TCF7L2* gene^47^,^48^. However, the molecular mechanisms implicated remain unresolved. Whilst many studies have focused on the function of TCF7L2 in adult β-cells^49^,^50^,^51^,^52^, little is known with regards to the role of this factor during pancreatic development. Global deletion of the *Tcf7l2* gene in the mouse leads to perinatal mortality due to hepatic alterations, but endocrine cell formation is apparently normal^53^. Likewise, conditional deletion of Tcf7l2 in the pancreas and in β-cells has no seeming impact on adult β-cell mass at least under normal physiological growth^53^,^54^,^55^. However, detailed characterization of endocrine differentiation in embryonic stages or neonatal endocrine cell counts was not provided in either study. Intriguingly, two recent investigations have shown that expression of dominant negative versions of Tcf7l2 in embryonic β-cells results in decreased β-cell mass and altered β-cell gene expression^56^,^57^. Our present findings provide additional support for a negative role of Tcf7l2 during pancreatic endocrine cell genesis that warrants further investigations. Tcf7l2 is a very complex protein that exhibits multiple isoforms and can act both as repressor or activator depending not only on the balance of the Tcf7l2 isoforms expressed at a given time and cell^58^, but also on post-translational modifications and available binding partners^58^,^59^,^60^. In this regard it is of note that Groucho/Tle proteins, which are recruited by TCF factors and mediate their repressor function, have been shown to be pivotal for endocrine differentiation downstream of Neurog3^61^. Further experiments aimed at the comprehensive analysis of the distribution of Tcf7l2 isoforms and the cellular context in which these variants are expressed in specific cell populations of the developing pancreas are needed to shed light into this issue.

In conclusion, this study places Wnt9a in the context of endocrine differentiation highlighting the notion that signalling ligands regulated by lineage-promoting factors may function as fine-tuners of developmental decisions promoted by these same factors. The present findings warrant further studies on the molecular circuitry governing Tcf7l2 activity in the embryonic pancreas and its potential implications for diabetes susceptibility. In addition, these data may also have important repercussions in our quest to improve β-cell differentiation protocols aimed at generating surrogate β-cells for transplantation purposes.

## Methods

### Mice

Mice were bred and maintained at the barrier animal facility of the University of Barcelona. Embryonic tissues were collected at indicated times, considering the morning of the appearance of a vaginal plug as embryonic day (E) 0.5. Principles of laboratory animal care were followed (European and local government guidelines) and animal procedures were approved by the Animal Research Committee of the University of Barcelona. Animals were euthanized by cervical dislocation. Mouse strains included *Wnt9a*^+/lacZ^ mice^16^, membrane-targeted tdTomato^62^ (Jackson Laboratory, Bar Harbor, ME, USA); *Pdx1*-Cre recombinase (Cre)^63^ and *Neurog3*-Cre^64^ (Jackson Laboratory). Mice were genotyped with primers provided in Supplementary Table S2.

### X-gal staining

Embryos were harvested and pancreas and gut were dissected and fixed for 1 h in fixative solution (2% PFA, 0.2% glutaraldehyde, 5 mM EGTA, 2 mM MgCl2 in 0.1 M phosphate pH7.3), washed in PBS, passed through 30% sucrose overnight and frozen in OCT. 15 μm cryosections were stained with X-gal solution containing detergent^65^ for 48 h at 37 °C.

### Pancreatic cell dispersion and flow cytometry

Pancreatic buds were harvested from E14.5-E15.5 embryos and treated with 0.125% trypsin-EDTA (Life Technologies) and 50 ng/ml Dnase I with agitation for 10–15 min at 37 °C. Digestion was inactivated by addition of RPMI-1640/10%FCS. Cells were recovered by centrifugation and resuspended in RPMI-1640/3%FCS for sorting using a BD FACSAria SORP machine.

### Cell culture and viral treatment

mPAC cells were grown in DMEM-4.5 g/L glucose (Sigma-Aldrich, St Louis, MO, USA) plus antibiotics supplemented with 10% fetal bovine serum. INS1E cells were cultured in RPMI-1640 medium supplemented with 10% fetal bovine serum, 10 mM Hepes, 2 mM L-glutamine, 1 mM sodium pyruvate and 50 μM β-mercaptoethanol. For adenoviral transduction experiments, cells were seeded onto 6-well plates and treated one day later with adenoviruses at a multiplicity of infection (moi) of 40 unless otherwise indicated for 2 h. Then, virus containing-media was replaced and cells were cultured for the indicated periods.

The coding sequence of the mouse *Wnt9a* cDNA was amplified from embryonic (E15.5) brain with the primers listed in Supplementary material and cloned into the adenoviral pAC.CMV shuttle vector. The recombinant adenovirus was constructed by homologous recombination in HEK293 cells. The adenovirus encoding human TCF7L2^53^ was kindly provided by Dr. C. Fillat (IDIBAPS, Barcelona, Spain). All other adenoviruses were previously described^21^,^65^.

### RNA isolation and RT-PCR analysis

Total RNA was isolated from cell lines or tissues using the RNeasy kit (Qiagen, Hilden, Germany) and from sorted cells using the NucleoSpin XS RNA kit (Mackerey-Nagel, Düren, Germany). First-strand cDNA was prepared using the Superscript III RT kit and random hexamer primers (Invitrogen, Carlsbad, CA, USA). Reverse transcription reaction was carried for 90 min at 50 °C and an additional 10 min at 55 °C. Real time PCR (qRT-PCR) was performed on an ABI Prism 7900 sequence detection system using SybrGreen reagents (Express Greener, Invitrogen). Primer sequences are provided in Supplementary Table S2.

### Transient transfections and luciferase assays

1.5 × 10^4^ mPAC cells were plated onto 96-well culture tissue plates one day before transfection. Transient transfections were performed using Metafectene (Biontex Laboratories GmbH, Martinsried, Germany) according to the manufacturer’s instructions. The amount of DNA used per well were: 250 ng of firefly luciferase reporter vectors, 2.5 ng of pRL.CMV and 5–20 ng of expression vector. Empty expression vector was added when necessary to keep the amount of DNA equal in all wells. Cells were harvested 48 h after transfection and luciferase activity was analyzed using the Dual-Luciferase Reporter Assay System (Promega) and a Veritas microplate luminometer (Promega). Luciferase readings were normalized to activities of the internal control vector pRL.CMV.

The mouse Wnt9a cDNA (see above) was cloned into the EcoRI/XbaI sites of the pCMV-TNT vector (Promega). The expression vectors (pCIG backbone) encoding a mutant form of β-catenin lacking aminoacids 29–48 and mouse Wnt3a were kindly provided by Dr. E.Martí (IBMB-CSIC, Barcelona, Spain). The luciferase reporter vectors Super8xTOPFlash and FOPFlash were kindly provided by Dr. R.T. Moon (University of Washington, Seatle, WA, USA). The Renilla luciferase reporter plasmid pRL-CMV was from Promega.

### Immunoblotting

Cells and embryonic pancreases were lysed in triple detergent lysis buffer (Tris-HCl 50 mM, NaCl 150 mM, 0.1% SDS, 1% NP40 and 0.5% Sodium Deoxycholate). 50 μg of lysates were separated by PAGE-SDS electrophoresis, transferred to a Polyscreen PVDF membrane (Perkin Elmer, Waltham, MA, USA) and incubated overnight at 4 °C with the antibodies indicated in Supplementary Table S3. Blots were visualized with ECL Reagent (Pierce Biotechnology, Rockford, IL, USA) using a LAS4000 Lumi-Imager (Fuji Photo Film, Valhalla, NY). Protein spots were quantitated with Image J software (http://rsb.info.nih.gov/ij/index.html).

### Immunofluorescence and morphometric analysis

Mouse embryos were fixed in 4% paraformaldehyde (PFA) for 3–6h. Tissues were subsequently washed, dehydrated, embedded in paraffin wax, and sectioned at 3 μm. For immunofluorescence, a standard immunodetection protocol was followed as described in^23^. Briefly, tissues were rehydrated and, when required, subjected to heat-mediated antigen retrieval in citrate buffer. After a blocking step in 5% donkey serum/ 0.2% Triton X-100, tissue sections were incubated overnight with primary antibodies and then for 1 h with secondary antibodies (Supplementary Table S3). Nuclei were stained with Hoechst 33258 (Sigma). Fluorescent images were captured using a Leica DMI 6000B widefield microscope or a Leica TCS SPE confocal microscope. For morphometrical analysis, total pancreas was sectioned at 3 μm and distributed as serial sections onto sets of 5 slides. At least 10 sections 45 μm apart per animal were analyzed using Image J software (http://rsb.info.nih.gov/ij/index.html).

INS1E cells were fixed in 4% PFA for 20 min at room temperature and permeabilized in PBS with 0.2% Triton X-100. After a blocking step of 1 h in 3% normal donkey serum, cells were incubated overnight with the indicated primary antibodies. After washes, cells were incubated with the secondary antibodies for 1 h at RT and. nuclei were stained for 3 min in a 1:500 dilution of Hoechst 33258 (Sigma).

### Statistical analysis

Data are presented as mean ± standard error of the mean (SEM). Statistical significance was tested using Student’s t-test.

## Additional Information

**How to cite this article**: Pujadas, G. *et al. Wnt9a* deficiency discloses a repressive role of Tcf7l2 on endocrine differentiation in the embryonic pancreas. *Sci. Rep.* **6**, 19223; doi: 10.1038/srep19223 (2016).

## Supplementary Material

Supplementary Information
