# Supplementary material for: Wnt9a deficiency discloses a repressive role of Tcf7l2 on endocrine differentiation in the embryonic pancreas
Source: Sci Rep. 2016 Jan 14;6:19223. doi: 10.1038/srep19223 (PMC4725895; doi:10.1038/srep19223)
Supplement: Supplementary Information [file srep19223-s1.pdf]

SUPPLEMENTARY INFORMATION

Wnt9a deficiency discloses a repressive role of Tcf7l2 on endocrine differentiation in the embryonic pancreas

G. Pujadas[1,2], S. Cervantes[1,2], A. Tutusaus[1], M. Ejarque[1,2], L. Sanchez[1], A. García[1,2], Y. Esteban[1,2], L. Fargas[3], B. Alsina[3], C. Hartmann[4], R. Gomis[1,2,5], R. Gasa[1,2]

[1]Diabetes and Obesity Research Laboratory, Institut d'Investigations Biomediques August Pi i Sunyer, Barcelona, Spain.

[2]Centro de Investigación Biomédica en Red de Diabetes y Enfermedades Metabólica Asociadas, Barcelona, Spain.

[3]Departament de Ciències Experimentals i de la Salut, Facultat de la Salut i de la Vida, Universitat Pompeu Fabra, Barcelona, Spain.

[4] Dept. of Bone and Skeletal Research, Institute of Experimental Musculoskeletal Medicine (IEMM), University Hospital Muenster, Muenster, Germany.

[5] University of Barcelona, Barcelona, Spain.

Pujadas_Fig S1

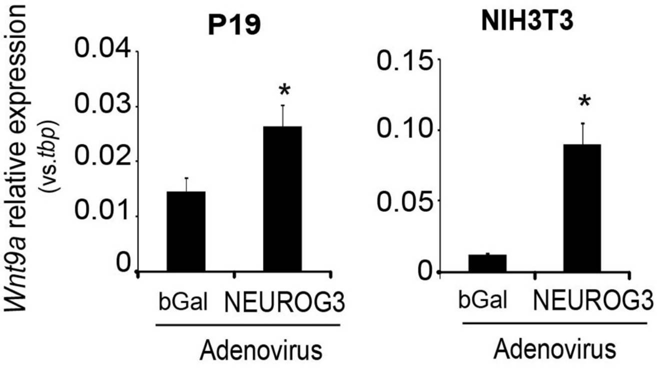

Figure S1. Activation of the *Wnt9a* gene in response to Neurog3. Mouse teratocarcinoma P19 cells and NIH3T3 fibroblasts were treated with the indicated adenoviruses and total cellular RNA was extracted 48h after virus treatment. *Wnt9a* mRNA levels were quantitated by qRT-PCR and expressed relative to the tbp gene expression. Bars represent mean ± SEM for at least 3 independent determinations per cell line. *$p<0.05$ vs. Ad-bGal.

Pujadas_Fig S2

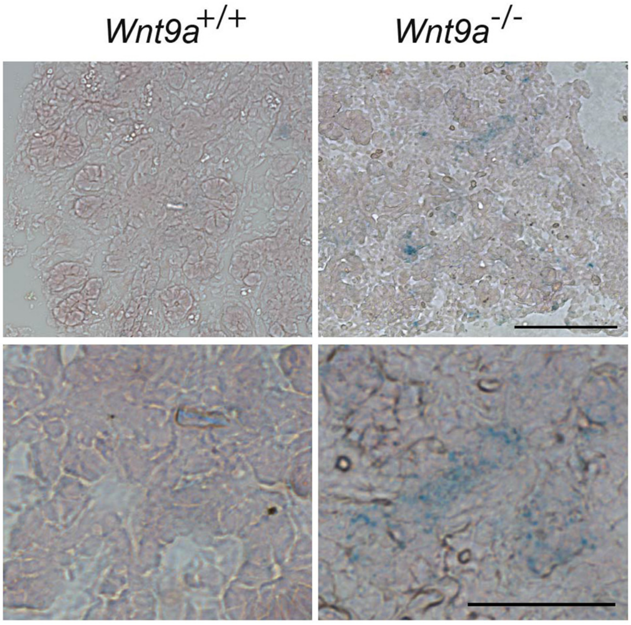

Figure S2. X-gal staining of pancreatic cryosections from E14.5 *Wnt9a*$^{+/+}$ and *Wnt9a*$^{-/-}$ embryos. Scale bars represent 100 µm (top row) and 60 µm (bottom row).

Pujadas_Fig S3

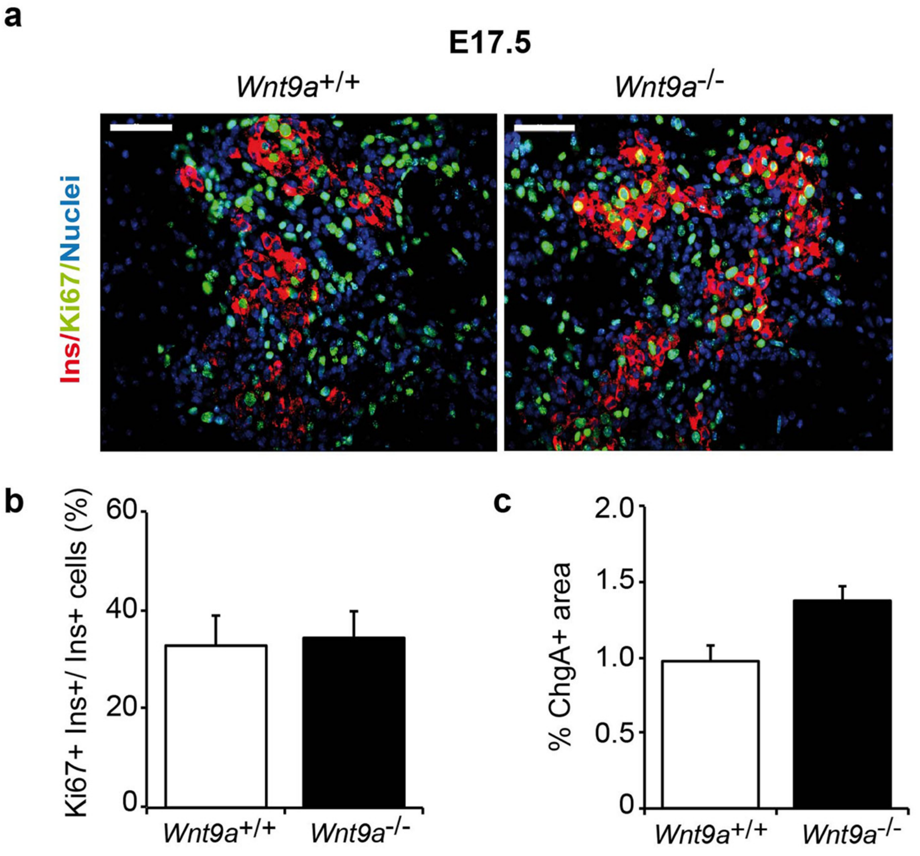

Figure S3. β-cell proliferation at E17.5. (a) Inmunofluorescense staining of pancretic tissue from Wnt9a$^{+/+}$ and Wnt9a$^{-/-}$ embryos at E17.5 with antibodies againts insulin (red) and the proliferation marker ki67 (green). Nuclei are labelled with Hoescht (blue). **(b)** Percentage of ki67-positive cells among insulin-positive cells. A total of 2500-3000 insulin-positive cells were counted per animal. **(c)** Percentage of ChromograninA+ area (as a measure of endocrine area) per total pancreatic area. Bars represent mean ± SD from 2 Wnt9a$^{+/+}$ and 2 Wnt9a$^{-/-}$ embryos. Scale bar is 50 µm.

Pujadas_ Fig S4

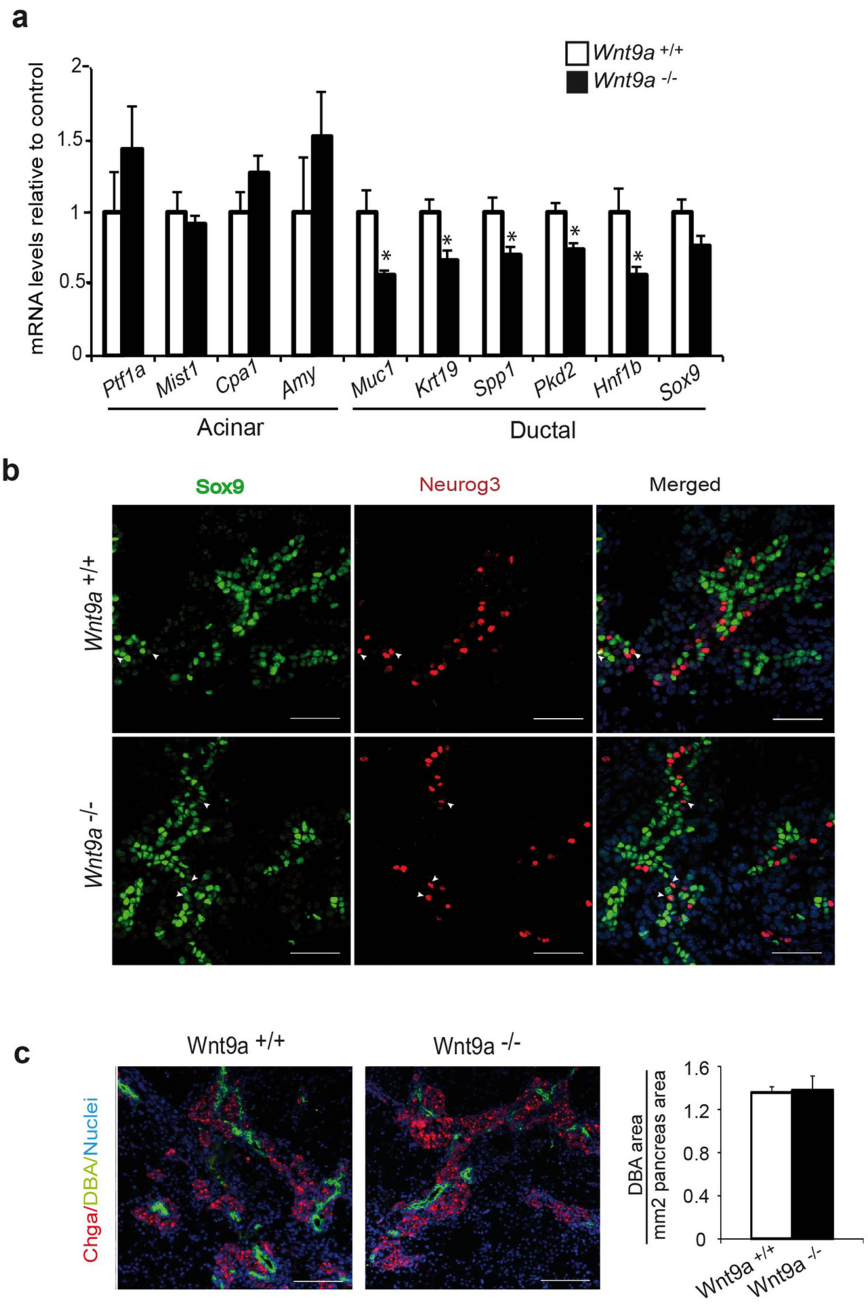

Figure S4. Characterization of the pancreatic exocrine compartment at E15.5. (a) Total RNA was isolated from pancreases of E15.5 Wnt9a$^{+/+}$ and Wnt9a$^{-/-}$ embryos. mRNA levels for acinar and ductal gene markers were quantitated by qRT-PCR and normalized against *tbp*. Results are expressed as fold relative to levels in Wnt9a+/+ pancreases (value of 1). Bars represent mean ± SEM for 8 Wnt9a$^{+/+}$ and 7 Wnt9a$^{-/-}$ embryos. *p<0.05, **p<0.01 vs Wnt9a+/+. **(b)** Double immunostaining for Sox9 (green) and Neurog3 (red) on paraffin sections of pancreas from Wnt9a$^{+/+}$ and Wnt9a$^{-/-}$ embryos at E15.5. Arrow-heads indicate cells co-expressing Neurog3 and Sox9. Note that expression of these two transcription factors is mutually exclusive in most cells. No differences were observed in this distribution pattern between knockouts and controls **(c)** Double immunostaining for DBA (green) and Chga (red) on paraffin sections of pancreas from Wnt9a+/+ and Wnt9a-/- embryos at E17.5. Scale bar is 100 µm. Morphometric quantification of DBA staining. Results are calculated as DBA+ area per pancreatic area. Bars represent mean ± SEM for 2 animals per genotype.

Pujadas_Fig S5

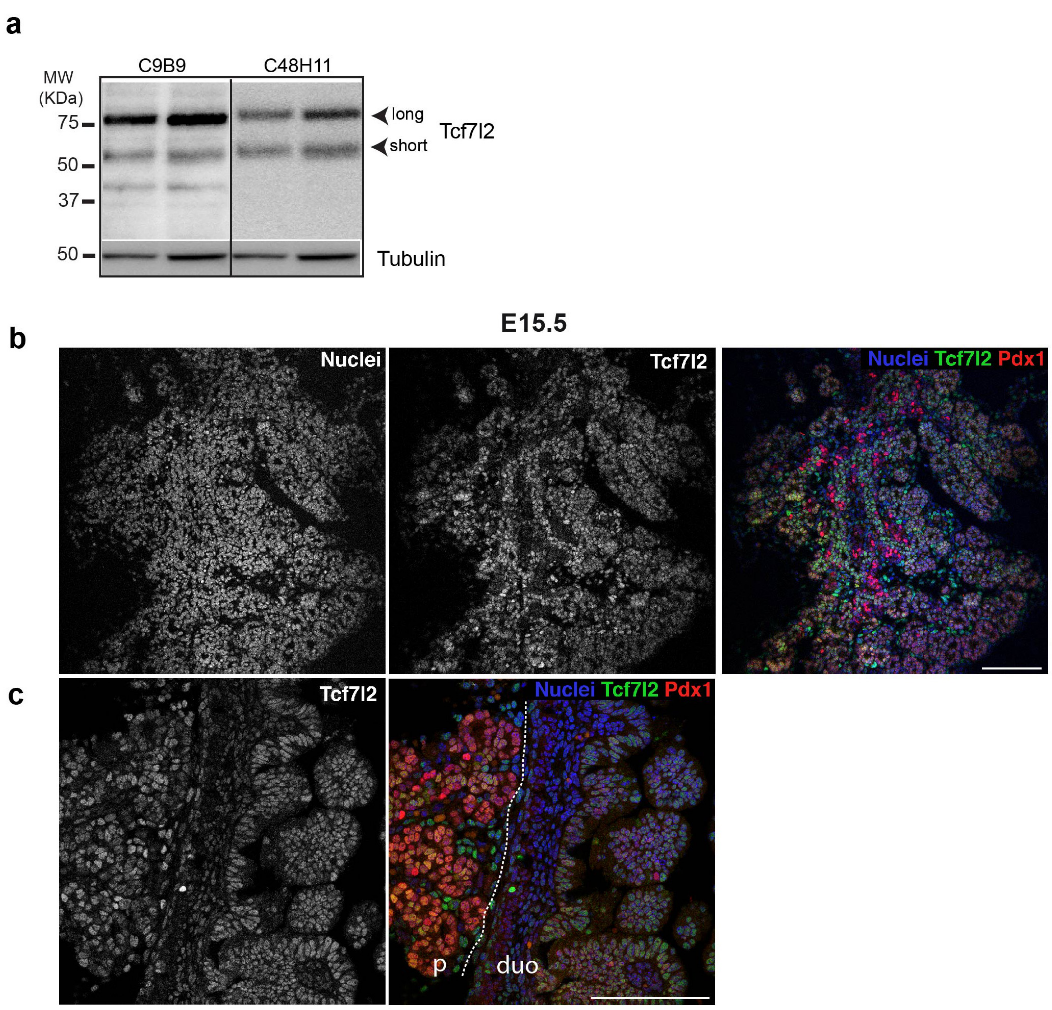

Figure S5. Distribution pattern of Tcf7l2 in the pancreas at E15.5. (a) Tcf7l2 protein expression was measured by immunoblot analysis using total pancreatic extracts from E15.5 mouse embryos and two different antibodies against Tcf7l2, C9B9 and C48H11. Tubulin was used as loading control. Representative images correspond to same membrane **(b)** Low magnification view of the pancreas shows broad expression of Tcf7l2 (antibody C48H11) in both trunk and tip epithelial regions, as well as in the Pdx1 negative mesenchyme. Some central areas corresponding to the endocrine compartment appear devoid of Tcf7l2. **(c)** View of the pancreas duodenum boundary revealed expression of the Tcf7l2 isoforms recognized by the C48H11 antibody in the epithelium of the duodenum. Scale bar is 100 μm.

Pujadas_Fig S6

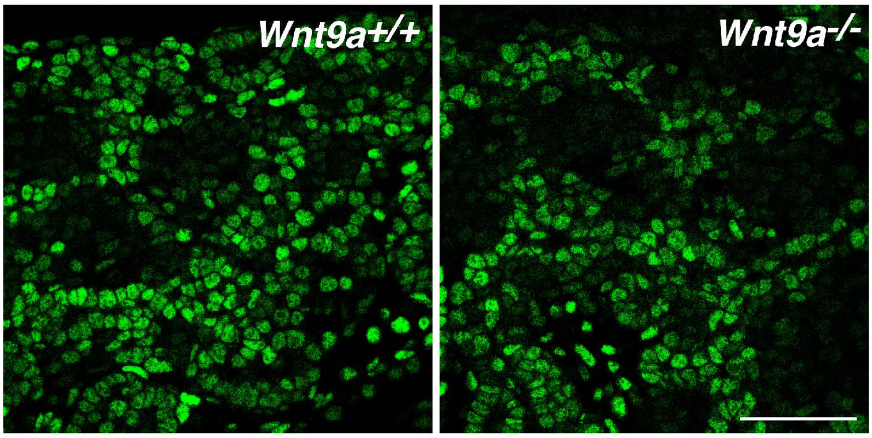

Figure S6. Distribution pattern of Tcf7l2 in E15.5 pancreases from *Wnt9a*$^{+/+}$ and *Wnt9a*$^{-/-}$ embryos. Immunostaining for Tcf7l2 with the C48H11 antibody in paraffin-fixed pancreatic sections from Wnt9a$^{+/+}$ and Wnt9a$^{-/-}$ embryos at E15.5. No obvious differences could be observed in overall levels or distribution between knockouts and controls. Scale bars represent 50 μm.

Table S1

GENE	SYMBOL	ROLE IN PATHWAY	REGULATED BY Neurog3	FC
**wingless-type MMTV integration site 9A**	Wnt9a	Ligand	Induced	1.50
**wingless-type MMTV integration site 7B**	Wnt7b	Ligand	Repressed	0.57
**frizzled homolog 6 (Drosophila)**	Fzd6	Receptor	Induced	1.60
**frizzled homolog 5 (Drosophila)**	Fzd5	Receptor	Repressed	0.63
**adenomatosis polyposis coli down-regulated 1**	Apcdd1	Intracellular component	Induced	2.16
**protein phosphatase 2 (formerly 2A). regulatory subunit A (PR 65). beta isoform**	Ppp2r1a	Intracellular component	Induced	1.42
**regulator of calcineurin 2**	Rcan2	Intracellular component non-canonical pathway	Induced	1.61
**Rho-associated coiled-coil containing protein kinase 2**	Rock2	Intracellular component non-canonical pathway	Repressed	0.80
**shisa homolog 2 (Tmem46)**	Shisa2	Modulator	Induced	2.40
**insulin-like growth factor binding protein 4**	Igfbp4	Modulator	Repressed	0.70
**leucine rich repeat containing G protein coupled receptor 5**	Lgr5	Modulator and target	Induced	1.58
**cadherin. EGF LAG seven-pass G-type receptor 1 (flamingo homolog. Drosophila)**	Celsr1	Modulator non-canonical pathway	Repressed	0.56
**microphthalmia-associated transcription factor**	Mitf	Nuclear modulator	Induced	1.58
**transducin (beta)-like 1X-linked receptor 1**	Tbl1xr1	Nuclear modulator	Repressed	0.80
**BMP and activin membrane-bound inhibitor. homolog (Xenopus laevis)**	Bambi	Modulator and target	Induced	2.76
**dickkopf homolog 1 (Xenopus laevis)**	Dkk1	Modulator and target	Repressed	0.43
**cyclin E2**	Ccne2	Target	Induced	2.52
**FBJ osteosarcoma oncogene**	c-Fos	Target	Repressed	0.65

Table S1. Wnt-related genes identified as potential targets of Neurog3 in mPAC cells. Microarray analysis identified several genes related to the Wnt signaling pathway that were differentially expressed between mPAC cells transduced with an adenovirus encoding Neurog3 (AdV-Neurog3) or with a control virus expressing beta-Galactosidase (AdV-Bgal). FC (Fold-Change) values refer to Neurog3/bGal expression ratio.

Table S2. List of oligonucleotides

GENE SYMBOL	SEQUENCE	APLICATION
Actb	5' TGAGAGGGAAATCGTGCGTG<br>3' TGCTTGCTGATCCACATCTGC	RT-PCR
Amy	5' TGGCGTCAAATCAGGAACATG<br>3' AAAGTGGCTGACAAAGCCCAG	qRT-PCR
Axin2	5' AAAACGGATTCAGGTCCTTCAA<br>3' GCAAAGACATAGCCGGAACCTA	qRT-PCR
Ccdn1	Mm00432359_m1 Taqman Gene Expression Assay	qRT-PCR
Chga	5' AGGGGACACCAAGGTGATGA<br>3' AGCAGATTCTGGTGTCGCAG	qRT-PCR
Chgb	5' GAATTGGGGATATGAGAAGAGAAGC<br>3' AGATCCATCGCAGCCAAGTTC	qRT-PCR
Cpa1	5' GCCACGGTAAGTTTCTGAGCA<br>3' ACACCCACAAAACGAATCGC	qRT-PCR
Ctnnb1	5' AGCTCGTGTCCTGTGAAGCCCG<br>3' TGTGCGCTGAGCTTCAGGTACC	qRT-PCR
Dkk1	5' AAACCTTGGTAATGACCACAACG<br>3' AGAAGTGTCTTGCACAACACA	qRT-PCR
Dll1	5' TGAGAGAGGAAGGGAGAGGAA<br>3' AGTGCAATGGGAACAACCAG	qRT-PCR
Gcg	5' AGGAATTCATTGCGTGGCTG<br>3' CAATGGCGACTTCTTCTGGG	qRT-PCR
Ghrl	5' CCATCTGCAGTTTGCTGCTA<br>3' GCTTGTCCTCTGTCCTCTGG	qRT-PCR
Hes1	5' TGCTACCCCAGCCAGTGTCAAC<br>3' TTCTTGCCCTTCGCCTCTTC	qRT-PCR
Hnf1b	5' GGCCGCGGTGACTCAGCTAC<br>3' CCAGGCTTGCAGTGGACACTGTTT	qRT-PCR
Iapp	5' CTCCAAACTGGCAGGTGTCC<br>3' TCCGTTTGTCCATCTGAGGG	qRT-PCR
Ins	5' AGCGTGGCTTCTTCTACACACC<br>3' CCAGCTCCAGTTGTGCCACT	qRT-PCR
Insm1	5' CTGGCGGCGTATCCGAATC<br>3' CCTGGCGACGGAACTTCTT	qRT-PCR
Jag1	5' AAGAGACAGGCAGGCGATCT<br>3' GGGATGCTTCCAACTTCACAC	qRT-PCR
Jag2	5' ACGAGGAGGATGAAGAGCTGA<br>3' GGGGTCTTTGGTGAACTTGTG	qRT-PCR
Krt19	5' GGTGCCACCATTGACAACTC<br>3' CTGCATCTCCAGGTCAGTC	qRT-PCR
Mist1	5' GCTGACCGCCACCATACTTAC<br>3' TGTGTAGAGTAGCGTTGCAGG	qRT-PCR
Mnx1	5' GTCTACTGCGGGCATGATCC<br>3' CACCTCAAAACGCTTGGGTC	qRT-PCR
Muc-1	5' GGCATTCGGGCTCCTTTCTT<br>3' TGGAGTGGTAGTCGATGCTAAG	qRT-PCR
NeuroD1	5' GGATCAATCTTCTCTTCCGGTG<br>3' TGCGAATGGCTATCGAAAGAC	qRT-PCR
NEUROG3 (transgene)	5' GGGTCCCTCTACTCCCCAGTCTCC<br>3' CTCAAGCAGGCGGAAAAGGTGG	qRT-PCR
Neurog3 (mouse)	5' TTCTCATCGGTACCCTTGCTG<br>3' GCAGACTCACCAGGAAGTATGG	qRT-PCR
Nkx2-2	5' GCCTCCAATACTCCCTGCAC<br>3' GTCATTGTCCGGTGACTCGT	qRT-PCR
Nkx6-1	5' TGGACAGCAAATCTTCGCCCTG<br>3' TGTTGTAATCGTCGTCATCCTC	qRT-PCR
Notch1	5' ACTACGGCCGGGTGTGACG<br>3' TGGTGGAGAGGCTGCTGTGTAGTG	qRT-PCR
Notch2	5' CCACCTGCCTGGATAAGATCG<br>3' CTGCCCGTTGTTCACACAC	qRT-PCR
Pax4	5' GAGTACCCTGCTCTTTTTGCC<br>3' ACTCGATTGATAGAGGACACACT	qRT-PCR

Pax6	5’ TACCAGTGTCTACCAGCCAAT<br>3’ TGCACGAGTATGAGGAGGTCT	qRT-PCR
Pdx1	5’ CCCCAGTTTACAAGCTCGCT<br>3’ CTCGGTTCCATTCGGGAAAGG	qRT-PCR
Pkd2	5’ TGACCTACGGCATGATGAGC<br>3’ GGAGCCTTCGGTGAACTTCC	qRT-PCR
Ppy	5’ ACCCAGGCGACTATGCGACACC<br>3’AGCTCCCTGCCTTCAGCTCCAG	qRT-PCR
Ptf1a	5’ ACAAGCCGCTAATGTGCGAGA<br>3’ TTGGAGAGGCGCTTTTCGT	qRT-PCR
Sox9	5’ CAGCGAACGCACATCAAGAC<br>3’ GCTGTAGGAGATCTGTTGCGG	qRT-PCR
Spp1	5’ CTCCTGGCTGAATTCTGAGGGACT<br>3’ AAGCTTCTTCTCCTCTGAGCTGC	qRT-PCR
Sst	5’ ACCCCAGACTCCGTCAGTTTC<br>3’ ATCATTCTCTGTCTGGTTGGGC	qRT-PCR
Tbp	5’ ACCCTTCACCAATGACTCCTATG<br>3’ ATGATGACTGCAGCAAATCGC	qRT-PCR
Tcf7	5’ GTTCACCCACCCATCCTTGA<br>3’ CCTTGGGTTCTGCCTGTGTT	qRT-PCR
Lef1	5’ TCCGAAATCATCCCAGCCAG<br>3’ GTGTTCTCTGGCCTTGTCGT	qRT-PCR
Tcf7l1	5’ AGAAGCCGCGCGACTATTTC<br>3’ GCTGCTCAGGTCTGGAATCA	qRT-PCR
Tcf7l2	5’ GCTGGTCTGCACGGGATAAC<br>3’ GGAAGCGAAAGGCAAGGATTTAG	qRT-PCR
Wnt5a	5’ CTTCCGCAAGGTGGGCGATGC<br>3’ TTGCACAGGCGTCCCTGCGTG	RT-PCR
Wnt7b	5’ CGCTACGGCATCGACTTTTCTC<br>3’ ATTCCAGCTTCATGCGGTCCTC	qRT-PCR/ RT-PCR
Wnt9a	5’ GGTGGGCAAGCACCTAAAAC<br>3’ GTACAAGCTCTGGTGTTCGGG	qRT-PCR/ RT-PCR
	5’ CGCCTTCTATCGCCTTCTTGACGAG (neo12)<br>5’ GCGGCCCAAGCACACTAC<br>3’ GGCACTCCAGGGCACTCAT	genotype
	5’ ACGAATTCATGCTGGATGGGTCCCTTCT<br>3’ CGTCTAGAGGTCAGCCCTTGCAGGTATAG	Cloning
Wnt9b	5’ AGAGGCTTTAAGGAGACGGC<br>3’ GGGGAGTCGTCACAAGTACAG	RT-PCR

Table S3. List of antibodies

WB: western blot; IF: immunofluorescence

PRIMARY ANTIBODIES	RAISED IN	DILUTION	SOURCE
Anti-alpha tubulin	mouse	1:1000 (WB)	Sigma- Aldrich
anti-TCF7L2 (C48H11)	rabbit mAb	1:1000 (WB)<br>1:200-1:400 (IF)	Cell Signalling Tech
Anti-TCF7L2 (C9B9)	rabbit mAb	1:1000 (WB)	Cell Signalling Tech
anti-chromogranin A	rabbit	1:200 (IF)	Thermo Scientific
anti-glucagon	mouse	1:1000 (IF)	Sigma-Aldrich
anti-insulin	guinea pig	1:1000 (IF)	DAKO, Glostrup, Denmark
anti-somatostatin	rabbit	1:500 (IF)	DAKO, Glostrup, Denmark
anti-Neurogenin3	mouse	1:2000 (IF	Developmental Studies Hybridoma Bank, Iowa City, IA, USA
anti-Pdx1	guinea pig	1:500 (IF)	Abcam, Cambridge, UK
anti-Sox9	rabbit	1:7000 (IF)	Millipore, Billerica, MA, USA
anti-Nkx2-2	mouse	1: 500 (IF)	Developmental Studies Hybridoma Bank, Iowa City, IA, USA
anti-Nkx6-1	mouse	1:500	Developmental Studies Hybridoma Bank, Iowa City, IA, USA
anti-Foxa2	goat	1:100	Santa Cruz
Anti-ki67	rabbit	1: 200 (IF)	Thermo Scientific

SECONDARY ANTIBODIES	RAISED IN	DILUTION	SOURCE
Anti-mouse IgG peroxidase	sheep	1/5000	GE Healthcare
Anti-rabbit IgG peroxidase	donkey	1/5000	GE Healthcare
Cy2 anti-guinea pig	donkey	1/500	Jackson ImmunoResearch, Suffolk, UK
Cy3 anti-guinea pig	donkey	1/500	Jackson ImmunoResearch, Suffolk, UK
Cy3 anti-mouse	goat	1/500	Jackson ImmunoResearch, Suffolk, UK

Cy3 anti-mouse	donkey	1/500	Jackson ImmunoResearch, Suffolk, UK
Cy2 anti-rabbit	donkey	1/500	Jackson ImmunoResearch, Suffolk, UK
Cy3 anti-rabbit	donkey	1/500	Jackson ImmunoResearch, Suffolk, UK
AMCA	mouse	1/500	Jackson ImmunoResearch, Suffolk, UK
AlexaFluor488	mouse	1/500	Jackson ImmunoResearch, Suffolk, UK

A FITC conjugate of the duct-binding lectin *Dolichos biflorus* agglutinin was purchased from Vector Laboratories (Burlingame, CA).
